# The efficiency of Nextera XT tagmentation depends on G and C bases in the binding motif leading to uneven coverage in bacterial species with low and neutral GC-content

**DOI:** 10.3389/fmicb.2022.944770

**Published:** 2022-07-14

**Authors:** Bo Segerman, Ásgeir Ástvaldsson, Linda Mustafa, Joakim Skarin, Hanna Skarin

**Affiliations:** ^1^Department of Microbiology, National Veterinary Institute (SVA), Uppsala, Sweden; ^2^Department of Medical Biochemistry and Microbiology, Uppsala University, Uppsala, Sweden; ^3^Department of Biology, Swedish Food Agency, Uppsala, Sweden

**Keywords:** Nextera XT, uneven, coverage, GC, bacterial, genome, *Campylobacter*

## Abstract

Whole-genome sequencing (WGS) is becoming the new standard for bacterial high-resolution typing and the performance of laboratories is being evaluated in interlaboratory comparisons. The use of the Illumina Nextera XT library preparation kit has been found to be associated with poorer performance due to a GC-content-dependent coverage bias. The bias is especially strong when sequencing low GC-content species. Here, we have made an in-depth analysis of the Nextera XT coverage bias problem using data from a proficiency test of the low GC-content species *Campylobacter jejuni*. We have compared Nextera XT with Nextera Flex/DNA Prep and examined the consequences on downstream WGS analysis when using different quantities of raw data. We have also analyzed how the coverage bias relates to differential usage of tagmentation cleavage sites. We found that the tagmentation site was characterized by a symmetrical motif with a central AT-rich region surrounded by Gs and Cs. The Gs and Cs appeared to be the main determinant for cleavage efficiency and the genomic regions that were associated with low coverage only contained low-efficiency cleavage sites. This explains why low GC-content genomes and regions are more subjected to coverage bias. We furthermore extended our analysis to other datasets representing other bacterial species. We visualized how the coverage bias was large in low GC-content species such as *C. jejuni*, *C. coli*, *Staphylococcus aureus*, and *Listeria monocytogenes*, whereas species with neutral GC-content such as *Salmonella enterica* and *Escherichia coli* were only affected in certain regions. Species with high GC-content such as *Mycobacterium tuberculosis* and *Pseudomonas aeruginosa* were hardly affected at all. The coverage bias associated with Nextera XT was not found when Nextera Flex/DNA Prep had been used.

## Introduction

Whole-genome sequencing (WGS) is being implemented as a high-resolution typing method in surveillance and outbreak investigations of pathogenic bacteria. Information collected through surveys, proficiency tests (PTs), and other interlaboratory comparisons points at a development where Illumina sequencing has become the most common sequencing technology in use for bacterial WGS typing (Eurl-Campylobacter, 2020; Eurl-Vtec, 2020; Uelze et al., 2020a). It has become apparent that the choice of method for the next-generation sequencing (NGS) library preparation step can have an impact on the quality of WGS analysis. Especially, the Illumina Nextera XT library preparation kit (hereafter only referred to as Nextera XT) has been reported to be associated with reduced quality in downstream analysis for several different NGS applications, including WGS and metagenomics (Lan et al., 2015; Tyler et al., 2016; Grutzke et al., 2019; Sato et al., 2019; Seth-Smith et al., 2019; Uelze et al., 2020a,b). The reason appears to be due to uneven sequencing depth/coverage over the target sequence (coverage bias), which appears to be connected to the GC-content. Genomic regions having both high and low GC-content have been associated with low sequencing coverage (Tyler et al., 2016; Sato et al., 2019; Seth-Smith et al., 2019). Despite the reported quality problems, Nextera XT is still used by many laboratories (Eurl-Campylobacter, 2020; Uelze et al., 2020a). The aim of this paper was to obtain a better understanding of the problem associated with Nextera XT from a bacterial WGS perspective.

The Nextera technology was first launched by Epicentre Biotechnologies but was acquired by Illumina in 2011. Nextera is based on “tagmentation,” where DNA fragmentation and adaptor tagging of the fragments are done in the same step (Marine et al., 2011). This has been made possible by loading the transposase Tn5 with a pair of adaptor sequences instead of the transposon sequence that is normally carried by Tn5. A Nextera version for small genomes, amplicons, and plasmids named Nextera XT was also introduced. In 2017, a new version of Nextera called Nextera DNA Flex was released. In this new version, the Tn5 transposase had been immobilized on beads, which allowed for easier normalization between samples and a broader DNA input range (Bruinsma et al., 2018). The new kit was also claimed to give a more even sequence coverage. Nextera DNA Flex was recently renamed Illumina DNA Prep (hereafter referred to as Nextera Flex/DNA Prep). The improved coverage distribution of Nextera Flex/DNA Prep has been confirmed in several studies (Grutzke et al., 2019; Sato et al., 2019; Seth-Smith et al., 2019).

Strong evidence has accumulated that Nextera XT is associated with bias in the genomic or metagenomic sequence coverage and that there is a connection to the GC-content (Lan et al., 2015; Tyler et al., 2016; Grutzke et al., 2019; Sato et al., 2019; Seth-Smith et al., 2019; Uelze et al., 2020a,b). Genomes with low GC-content seem to be more affected than genomes with more neutral or higher GC-content, but also regions with high GC-content have been pointed out as giving rise to low coverage. It is not fully understood what is causing these coverage fluctuations, but it is likely connected to bias in the Tn5 transposase tagmentation reaction. Tn5 prefers certain motifs, and this can be seen as a distorted base composition in approximately the first 10 bases of the sequence reads generated when sequencing with Nextera-based kits. We hypothesize that the reasons for the coverage bias introduced by Nextera XT can be better understood if the bias is studied on the level of tagmentation sites instead of read coverage as done in previous studies.

Thus, we have made an in-depth analysis of the bias introduced by Nextera XT, its consequences on downstream WGS analysis, and an analysis of tagmentation site usage. We have used a dataset from a PT on WGS of the low GC-content species *Campylobacter jejuni* (Eurl-Campylobacter, 2020). The dataset includes sequence data generated using both Nextera XT and Nextera Flex/DNA Prep. We also extend the context of our conclusions by using additional datasets of other bacterial species representing low, medium, and high GC-content.

## Materials and methods

### Datasets used in the analysis

In this study, a reference genome refers to a completed or nearly completed genome sequence of the same isolate/strain sequenced with the Nextera XT or Nextera Flex/DNA Prep kits, except in the SNP analysis where the reference used was the reference genome of the species *C. jejuni*, from strain NCTC 11168 (NC002163.1). The data from the EU reference laboratory (EURL) for *Campylobacter* PT 28 are found under BioProject PRJEB45600. The *C. jejuni* reference genome has accession GCA_912579705 and *C. coli* reference genome GCA_912579715. They have been assembled to the level of completed genomes using a combination of Illumina sequencing and Oxford Nanopore long read sequencing (Eurl-Campylobacter, 2020). The *C. jejuni* reference genome consists of a single 1.8 Mb (1,805,160 bases) chromosome with an average GC-content of 30.29%. The *C. coli* reference genome consists of a 1,811,988 bp chromosome and a 104,639 bp plasmid with an average GC-content of 31.18%. The external datasets were obtained from Uelze et al. with BioProject accession PRJEB37768 and PRJNA638266 where data from Lab4 and Lab6 were used (Uelze et al., 2020a), from Sato et al. with BioProject accession PRJDB8030 (Sato et al., 2019) and from Seth-Smith et al. with BioProject accession PRJEB31421 (Seth-Smith et al., 2019). Reference sequences for the Sato et al. datasets were NC_000913.3 (*Escherichia coli* K-12, 50.79% GC-content); NC_002695.2, NC_002127.1, and NC_002128.1 (*E. coli* O157:H7 Sakai, 50.48% GC-content); NC_003923.1 (*Staphylococcus aureus* MW2, 32.83% GC-content); and NC_002745.2 (*S. aureus* N315, 32.84% GC-content). Reference sequences for the Seth-Smith et al. dataset were AE009951.2 (*Fusobacterium nucleatum* ATCC 25586, 27.15% GC-content); NC_002163.1 (*C. jejuni* NCTC 11168, 30.55% GC-content); NZ_CP009361.1 and NZ_CP009362.1 (*S. aureus* ATCC 25923, 32.86% GC content); NZ_CP008814.1, NZ_CP008815.1, and NZ_CP008816.1 (*Enterococcus faecalis* ATCC 29212, 37.35% GC-content); NZ_CP008926.1 (*Streptococcus pyogenes* ATCC 19615, 38.48% GC-content); NZ_014370.1 and NZ_014371.1 (*Prevotella melaninogenica* ATCC 25845, 40.98% GC-content); CP009072.1 (*E. coli* ATCC 25922, 50.54% GC-content); NZ_CP014696.2, NZ_CP014697.2, and NZ_CP014698.2 (*Klebsiella quasipneumoniae* ATCC 700603, 57.73% GC-content); NC_009525.1 (*Mycobacterium tuberculosis* H37Ra, 65.61% GC-content); CP015117.1 (*Pseudomonas aeruginosa* ATCC 27853, 66.11% GC-content); NZ_CP016442.1, NZ_CP016443.1, and NZ_CP016444.1 (*Burkholderia stabilis* ATCC BAA-67, 66.42% GC-content); and CP001628.1 (*Micrococcus luteus* NCTC 2665, 73.00% GC-content).

### The EURL-*Campylobacter* proficiency test number 28

A detailed description of the EURL-*Campylobacter* PT number 28 can be found in the PT 28 report (Eurl-Campylobacter, 2020). In brief, PT 28 was organized by the EURL-*Campylobacter* in 2020 with the aim to compare the quality of whole-genome sequencing data generated at different national reference laboratories (NRLs) for *Campylobacter*. In the PT, strains from two *Campylobacter* species were used, one *C. jejuni* strain and one *C. coli* strain. Sequencing was done by the participating laboratories, both from provided DNA samples and from DNA prepared by each participant from a fresh culture of each of the provided freeze-dried bacterial strains.

In this study, we have made an in-depth analysis on the sequencing data from PT sample number 1, which was DNA from a *C. jejuni* clinical strain. Nine participating laboratories using Nextera XT or Nextera Flex/DNA Prep (including some laboratories not included in the PT 28 report due to late submission of data/results) gave permission for data to be used in this study. A summary of the data used in this study is shown in Table 1. Four laboratories used Nextera XT and five Nextera Flex/DNA Prep. The DNA was prepared by the EURL*-Campylobacter*. Strains were cultivated on horse blood agar and DNA was extracted using a Qiagen EZ1 robot and a Qiagen EZ1 DNA tissue kit (Qiagen, Hilden, Germany). The DNA was stabilized using Biomatrica DNAstable plus solution (Biomatrica, San Diego, CA, United States). Lab18 used Nextera XT and sequenced on an Illumina MiSeq using a MiSeq V3 kit (2 × 300 bp). Lab19 used Nextera Flex/Illumina DNA prep and sequenced on an Illumina MiSeq using a MiSeq V3 kit (2 × 150 bp). Lab20 used Nextera XT and sequenced on an Illumina NovaSeq (2 × 150 bp). Lab23 used Nextera Flex/Illumina DNA prep and sequenced on an Illumina MiSeq using a MiSeq V3 kit (2 × 300 bp). Lab24 used Nextera XT and sequenced on an Illumina MiSeq using a MiSeq V3 kit (2 × 300 bp). Lab49 used Nextera Flex/Illumina DNA prep and sequenced on an Illumina MiSeq using a MiSeq V3 kit (2 × 300 bp). Lab54 used Nextera XT and sequenced on an Illumina HiSeq2500 (2 × 100 bp). Lab58 used Nextera Flex/Illumina DNA prep and sequenced on an Illumina MiniSeq with a high output kit (2 × 150 bp). Lab61 used Nextera XT and sequenced on an Illumina MiSeq using a MiSeq V2 kit (2 × 250 bp).

**Table 1:** Overview of the partial dataset from EURL-*Campylobacter* proficiency test number 28 analyzed in this study.

Lab ID	Library kit	Machine	Read pairs	Read length	Coverage
L18	XT	MiSeq	274 k	2 × 300 bp	62X
L19	Flex/DNA Prep	MiSeq	540 k	2 × 150 bp	79X
L20	XT	NovaSeq	8,137 k	2 × 150 bp	1,116X
L23	Flex/DNA Prep	MiSeq	1,100 k	2 × 300 bp	305X
L24	XT	MiSeq	959 k	2 × 300 bp	264X
L49	Flex/DNA Prep	MiSeq	502 k	2 × 300 bp	140X
L54	XT	HiSeq	1,752 k	2 × 100 bp	185X
L58	Flex/DNA Prep	MiniSeq	460 k	2 × 150 bp	75X
L61	XT	MiSeq	307 k	2 × 250 bp	52X

### Read trimming and assembly

Reads from all laboratories were trimmed with Trimmomatic (Bolger et al., 2014), version 0.39, with following trimming parameters “ILLUMINACLIP:pathtoadapters/NexteraPE-PE.fa:2:30:10:2:true LEADING:3 TRAILING:3 SLIDING WINDOW:4:15 MINLEN:36.” Reads that were unpaired after trimming were discarded. Between 0.1% and 9% (on average 2%) of the read-pairs were lost during trimming. Data downsamplings were made with the tngs tool,^1^ version 2022.03.04. Trimmed reads were downsampled into titration series corresponding to 10X, 15X, 20X, 25X, 30X, 35X, 40X, 45X 50X, 60X, 70X, 80X, 90X, and 100X. Some datasets were not large enough to reach 100X. Trimmed and downsampled data were assembled with SPAdes (Bankevich et al., 2012), version 3.15.3, using default settings and including the “-isolate” option. Contigs shorter than 200 bp and contigs having 10% or less *k*-mer coverage compared to the average genomic *k*-mer coverage were filtered using the tngs tool. This step was added to remove contigs from contaminating species/contaminations due to traces of carry over between sequencing runs. Assembly quality metrics and reference genome coverage were determined using the tngs tool.

### Annotation and genome comparisons

Genes were predicted by using the annotation pipeline Prokka (Seemann, 2014), which depend on Prodigal (Hyatt et al., 2010) for gene prediction. Core genome multi-locus sequence typing (cgMLST) was made using Ridom SeqSphere+, version 8.2.0, using the Oxford v.1 schema (Cody et al., 2017). Single-nucleotide polymorphism (SNP) analysis was made using Snippy,^2^ version 4.0.2. The reference genome used for the SNP analysis was the GenBank *C. jejuni* reference NC002163.1. The percentage of the reference genome *k*-mers (20-mers) found in the assemblies was quantified using the tngs tool.

### Tagmentation site analysis

Tagmentation sites were determined by identifying the read start positions by mapping the reads to the reference genome. Trimmed reads were mapped with bowtie2 (Langmead and Salzberg, 2012), version 2.4.5, with default parameters. SAMtools (Li et al., 2009), version 1.14, was used with default settings to convert between SAM and BAM formats, to sort the alignments, to discard unaligned reads, to separate forward and reversed mapped reads, and to create files in mpileup format. The mpileup command was run with the-a option to include all coordinates, including those with zero depth. Coverage diagrams were made from the mpileup files. Tagmentation sites and their consensus sequences were determined using a custom Perl script that integrated the coverage information from the mpileup file and the read mapping information from two SAM files containing forward and reversed mapped reads, respectively. Only reads mapped with a full-length alignment having 100% identity were used in the analysis (determined by the CIGAR string in the SAM file and by comparing the alignment length to the read length).

### Graphs, heatmaps, and statistics

Graphs were plotted using GraphPad Prism, version 5. Pearson’s correlation coefficient calculations and the heatmap were made in Microsoft Excel. Correlation strength was classified using the criterion of Evans (1996). WebLogos were created with WebLogo 3, version 3.7.4, with the “composition” parameter set to the GC-content 30%.^3^ Statistical testing was made in GraphPad Prism, version 5 with unpaired *t*-tests. If the F-test indicated unequal variance, Welch’s correction was applied. The frequency bin data were too large to analyze in GraphPad Prism (~0.5 million datapoints per frequency bin) and were therefore tested using Microsoft Excel (Version 2,205).

## Results

### WGS quality parameters dependency on total amount of data

**Figure 1:**
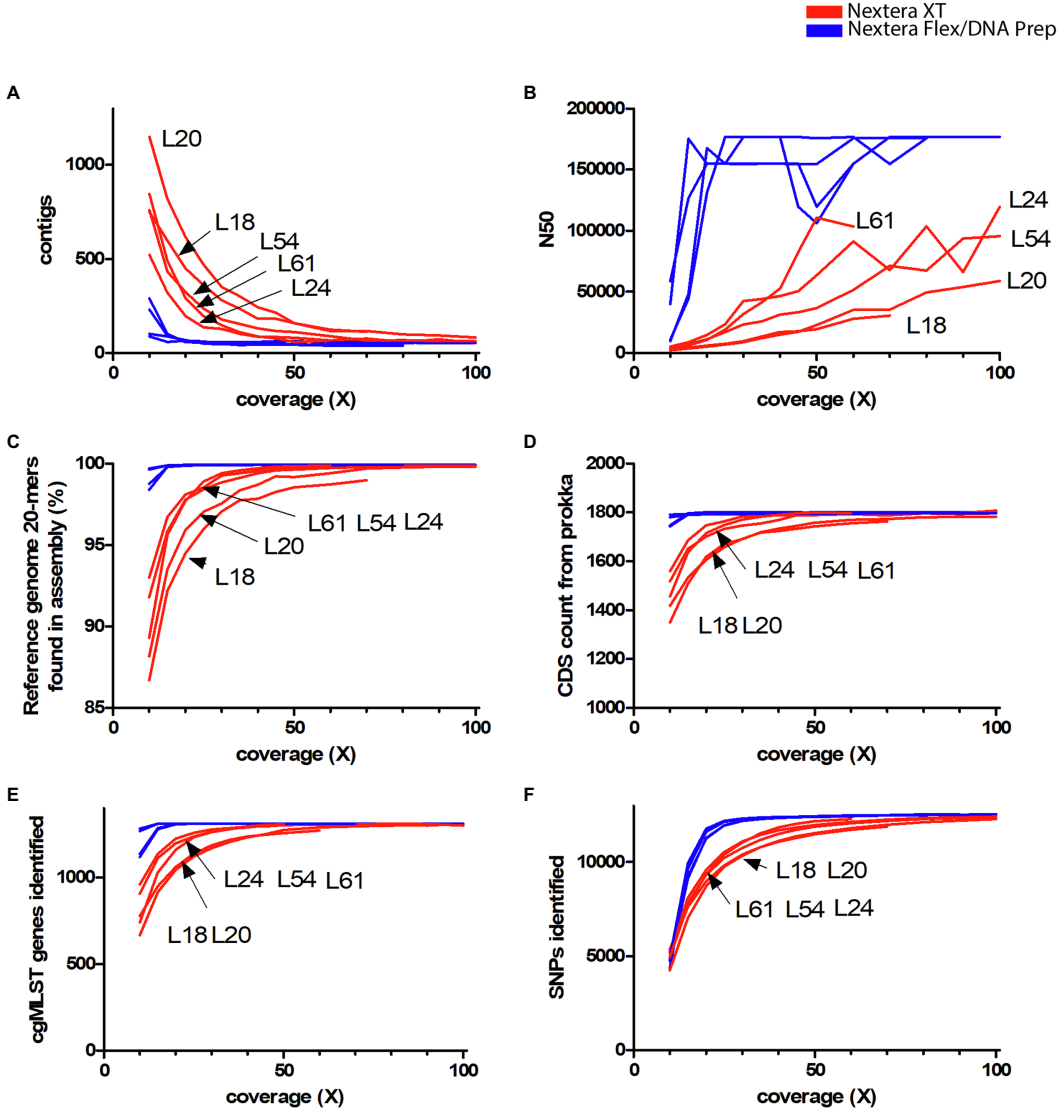
Dependency on the total amount of data used for quality parameters in the *Campylobacter jejuni* PT 28 dataset. The data have been colored to indicate the type of library prep kit used (Nextera XT red, Nextera Flex/DNA Prep blue). **(A)** The number of contigs generated by SPAdes assembler after filtering short and low coverage contigs. **(B)** N50 values of the assemblies. **(C)** The percentage of the *k*-mers present in the completed reference sequence found in the assemblies. **(D)** Number of genes found by the annotation tool Prokka. **(E)** Number of cgMLST genes (Oxford schema) called by Ridom SeqSphere+. **(F)** Number of SNPs called by Snippy against the reference genome NC002163.1.

Coverage bias can result in a lack of coverage in certain genomic regions. The more data that are used, the less genomic regions will lack coverage. Thus, the extent of the consequences of coverage bias depends on the total coverage. We therefore wanted to quantify the consequences of coverage bias and its dependency on the total amount of data used. Quality measures based on genome assembly, SNP, and gene calling were determined on titration series representing different average genomic coverages of the data from EURL-*Campylobacter* PT 28. The number of contigs obtained and the N50 values showed that assemblies generated with Nextera XT data had poor quality when less than 50X data were used (Figures 1A,B). The Nextera XT assemblies had lower quality than the Nextera Flex/DNA Prep assemblies also at higher coverages, but the difference was less pronounced. The difference between Nextera XT and Nextera Flex/DNA prep was most pronounced when N50 values were compared. There were some differences between the Nextera XT laboratories, with L20 and L18 generating the lowest quality values, but there was no obvious connection to factors such as read length or type of Illumina instrument used (Table 1). Laboratories using Nextera Flex/DNA Prep all reached high-quality assemblies already at 20X coverage (Figures 1A,B). The same was seen when looking at the percentage of the *k*-mers present in the completed reference genome sequence that the assemblies contained (Figure 1C). Similarly, the number of genes that could be called from the assemblies, both in total and when using a cgMLST schema, was strongly reduced in the assemblies generated with Nextera XT (Figures 1D,E). The most widely used methods for cluster analysis of bacterial WGS data are based on either cgMLST or SNP analysis and both lost a great deal of resolution when Nextera XT was used with a lower amount of input data (Figures 1E,F). The laboratories using Nextera XT needed approximately five times as much data to reach comparable levels of called cgMLST alleles or SNPs. Even at 100X coverage, slightly less genes and SNPs were called in data generated by Nextera XT compared to data generated with Nextera Flex/DNA Prep. The mean quality parameter values of the Nextera XT labs and the Nextera Flex/DNA Prep labs were compared at 25X and 50X coverage (*t*-tests). The differences were significant for all quality measures at 25X and for the number of contigs, N50 values, and SNPs called at 50X coverage (Table 2).

**Table 2:** *t*-tests comparing Nextera XT labs with Nextera Flex/DNA Prep labs for the quality control data from Figure 1 at coverages 25X and 50X.

	Value of *p* (25X)	Significant (25X)	Value of *p* (50X)	Significant (50X)
Contigs	0.0204	*	0.0473	*
N50	<0.0001	***	0.0074	**
Ref. 20-mers in assembly	0.0175	*	0.0873	not significant
CDS count	0.0193	*	0.1235	not significant
cgMLST genes	0.0157	*	0.0940	not significant
SNPs	<0.0001	***	0.0048	**

*p = 0.05; **p = 0.01 and ***p = 0.001.

### Coverage fluctuations and their relations to GC-content

**Figure 2:**
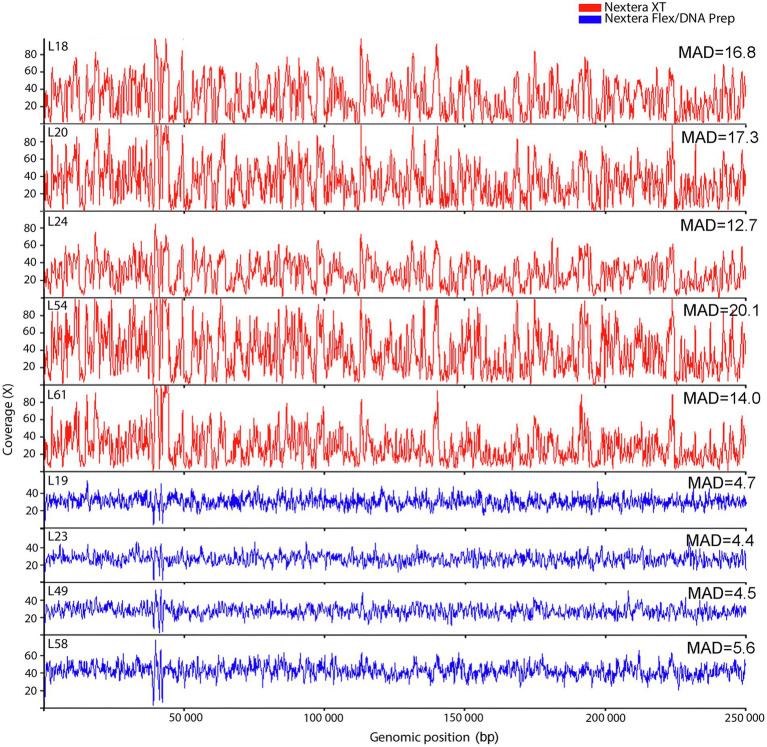
Coverage fluctuations along the first 250 kb of the reference genome for the laboratories in the *Campylobacter jejuni* PT 28 dataset. The data have been downsampled to equal depth and colored to indicate the type of library prep kit used (Nextera XT red, Nextera Flex/DNA Prep blue). MAD refer to Mean absolute deviation.

It is known from previous studies that the reason for the Nextera XT quality problems is connected to variation in coverage over the target sequence. To visualize this in our dataset, the coverage fluctuations over the first 250 kb of the reference genome were plotted using a sliding window approach (Figure 2). This gives a good visualization of how the Nextera XT data fluctuate between high and low coverage, whereas the Nextera Flex/DNA Prep data show more even coverage distributions. This is also seen when calculating the mean absolute deviation (MAD). Nextera XT had a significantly higher average MAD value than Nextera Flex/DNA Prep (*t*-test, *p* value 0.001). Visually, there appears to be a resemblance between the coverage curves from the different Nextera XT laboratories, suggesting the variation to be mainly sequence-dependent with only a small random component.

**Figure 3:**
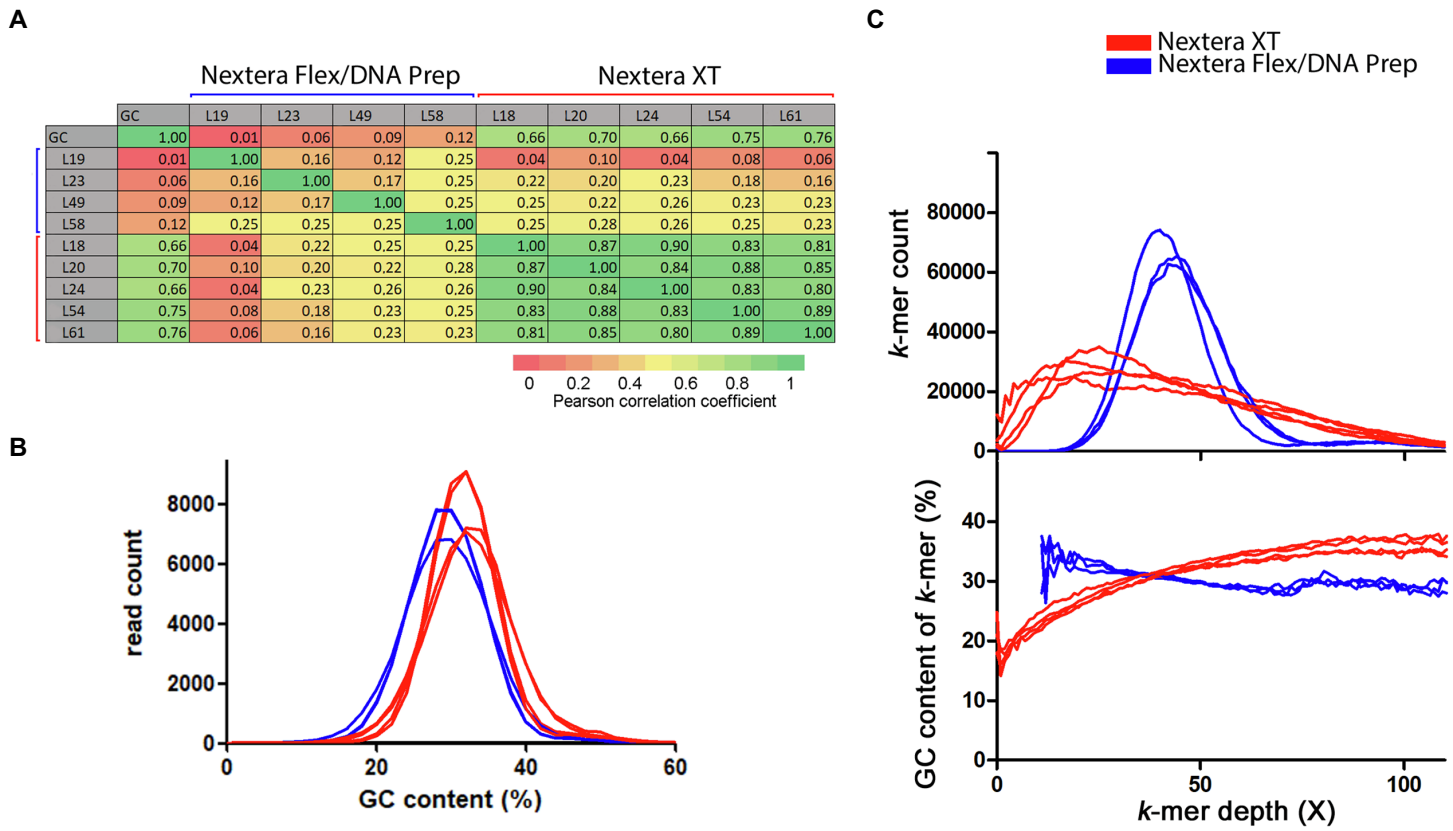
Connection between GC-content and coverage bias for the *Campylobacter jejuni* PT 28 dataset. The data have been colored to indicate the type of library prep kit used (Nextera XT red, Nextera Flex/DNA Prep blue). **(A)** Pearson’s correlation coefficients calculated between the sliding window coverage data and GC-content data. **(B)** Histogram over the GC-contents of the sequence reads. **(C)** Histogram over the depth of the reference sequence *k*-mers in the raw data downsampled to 50X (upper part) and the corresponding average GC-content of the *k*-mers in each histogram bin.

To further dissect the apparent similarity between the Nextera XT coverage curves and their connection to GC-content, Pearson’s correlation coefficients were calculated between the coverage data generated by the sliding window analysis from all laboratories and toward the GC-content calculated in the same sliding window analysis. A heatmap of the correlation coefficients shows that there was a very strong correlation between the coverage fluctuations for all Nextera XT laboratories (Figure 3A). The much less pronounced fluctuations found in the curves from laboratories using Nextera Flex/DNA Prep were weakly or very weakly correlated to each other and likely represented mostly random coverage fluctuations. The Nextera XT coverage curves also showed a strong positive correlation with the GC-content (Figure 3A), indicating that the low coverage was connected to low GC regions and high coverage with high GC regions.

When measuring GC-content on a per read-basis, the Nextera XT laboratories had a higher average GC-content compared to reads produced by the Nextera Flex/DNA Prep laboratories (Figure 3B). The difference between the mean values was significant (*t*-test, *p* value 0.0049). This is in line with the conclusion that Nextera XT underperforms in low GC regions, thereby giving an overestimation of the genomic GC-content. We made a histogram of the coverages of all 20-mers found in the reference genome sequence within the raw read data (Figure 3C). The Nextera XT laboratories had a much broader and asymmetrically shaped *k*-mer coverage distribution with a peak displaced toward lower coverage depth compared to the average coverage. The Nextera Flex/DNA Prep histograms had shapes more similar to a normal distribution. The average GC-content of the *k*-mers in each histogram bin was also plotted. For the Nextera data, this clearly showed that lower coverage was strongly connected to lower GC-content. The Nextera Flex/DNA Prep coverage showed much less connection to GC-content. To the contrary, the few genomic *k*-mers with low coverage present in data from Nextera Flex/DNA Prep were rather associated with a slightly increased GC-content. In summary, the data showed that Nextera XT results in major coverage dips affecting a large part of the sequenced genome and the regions with low coverage are characterized by a low GC-content. The Nextera Flex/DNA Prep gave coverage distributions that resemble a normal distribution as would be expected by an unbiased fragmentation.

### Analysis of low coverage regions and the tagmentation target sites

**Figure 4:**
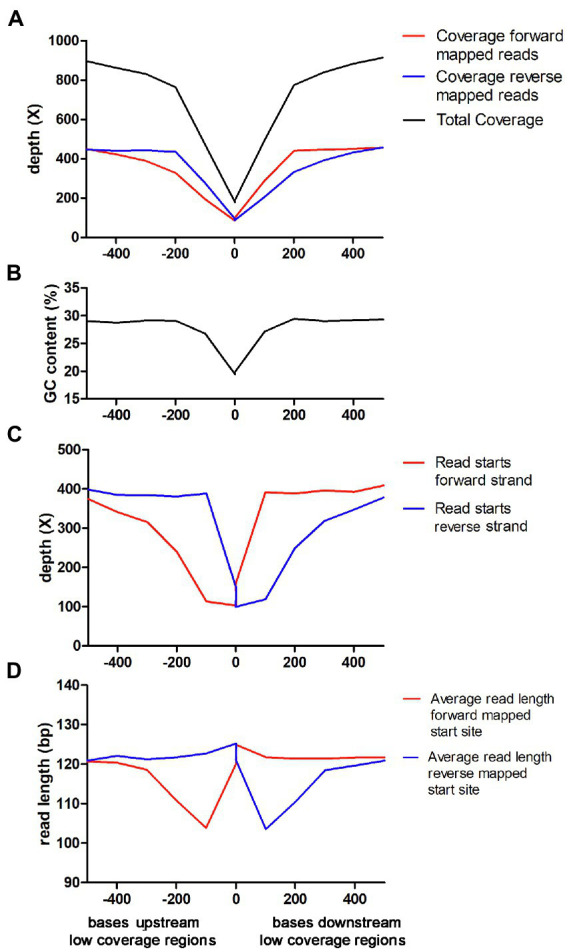
Characterization of low coverage regions for Nextera XT laboratories in the *Campylobacter jejuni* PT 28 dataset. Note that the X-axis is the same in all panels and the description is found in the lowest panel. **(A)** Average read depth of forward and reversed mapped reads upstream and downstream the low coverage regions. **(B)** GC-content upstream and downstream the low coverage regions. **(C)** Average number of read starts of forward and reversed mapped reads upstream and downstream the low coverage regions. **(D)** Average read length of forward and reversed mapped reads upstream and downstream the low coverage regions.

The results outlined above showed that the connection between low GC-content and low coverage is very strong in *C. jejuni* when using Nextera XT. To analyze this in-depth, we used the data from Lab 20, which had submitted a very large amount of sequencing data from an Illumina NovaSeq instrument. We used a sliding window analysis with a window size of 100 bp and marked windows as being “low coverage windows” if they had a coverage below 25% of the average. Directly adjacent low coverage windows were then merged into “low coverage regions.” Then, several parameters were analyzed relative to the start (upstream) and end (downstream) of the low coverage regions. Thus, the X-axis represents the distance measured in bp from all defined low coverage regions (upstream and downstream). The Y-axis represents the average properties of the sequence reads that were mapped in the 100 bp window beginning at the distance specified by the X-axis from any low coverage region. The analysis confirmed that the total coverage was highly connected to the GC-content (Figures 4A,B). When the coverage was divided into forward and reverse strand coverage, it became evident that the forward strand coverage was comparatively lower directly upstream the low coverage regions and the reverse strand coverage was comparatively lower directly downstream the low coverage region (Figure 4A). The forward strand starts of the sequence reads showed a pronounced lowered frequency upstream of the low coverage regions and likewise downstream for the low overage regions (Figure 4C). Thus, the fragments that were expected to end within the low coverage/low GC region appeared to be reduced. Furthermore, the average read length of the reads starting in forward direction directly upstream the low coverage regions were shorter than expected and likewise for the reads starting in reverse direction directly downstream the low coverage regions (Figure 4D). In summary, the reads starting in positions where an expected fragment ending would be situated in the low coverage regions were not only reduced in numbers but were also often shorter and ended before the low coverage region.

**Figure 5:**
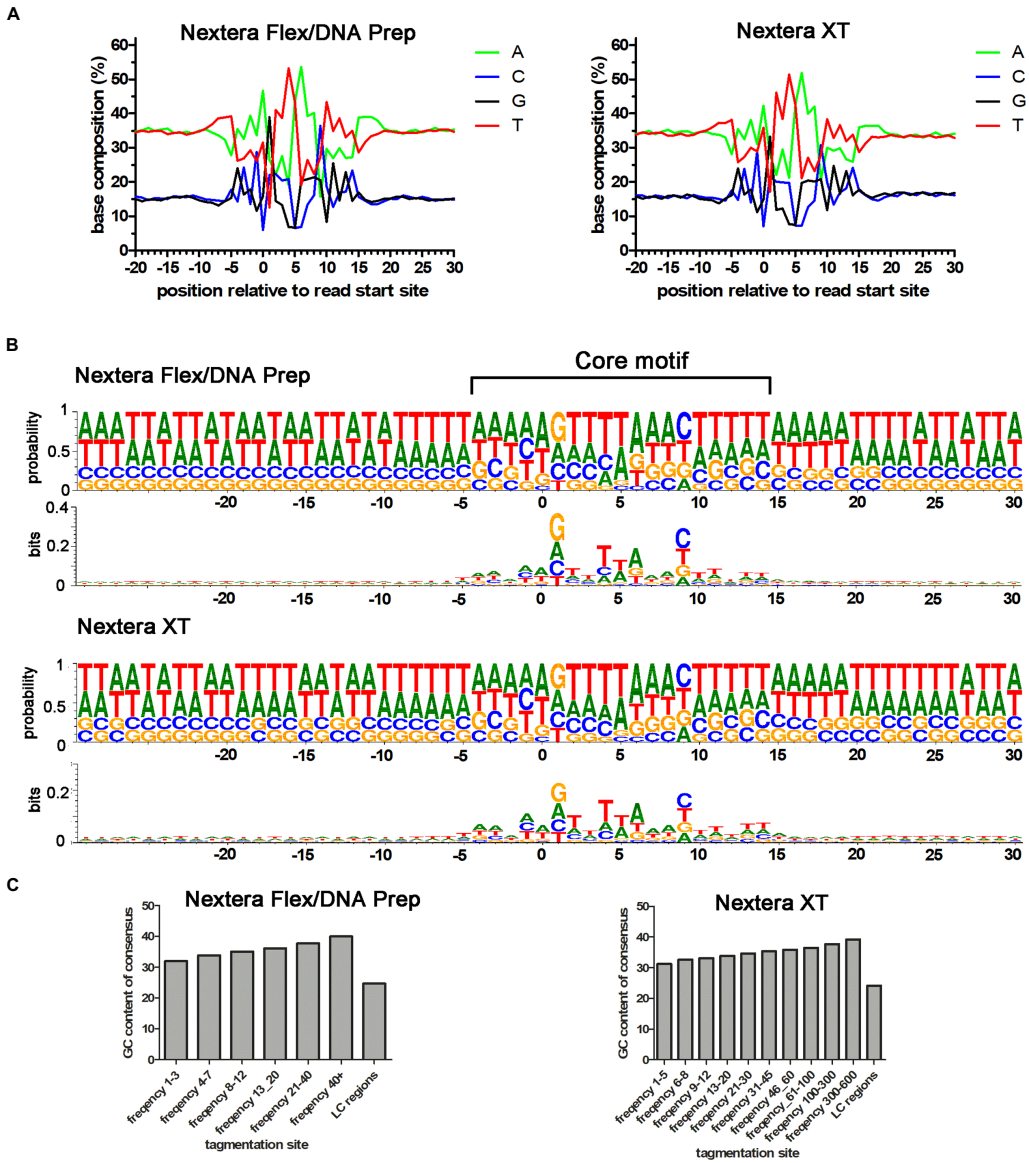
Base composition of Nextera tagmentation sites. **(A)** Average base composition of tagmentation sites relative to the read start position for the Nextera XT and Nextera Flex/DNA Prep data in the *Campylobacter jejuni* PT 28 dataset. **(B)** DNA Logo plot of the data shown in A. Both probability and bits indicating the importance of individual positions are plotted. **(C)** GC-content of the tagmentation sites when divided into groups of different cleavage efficiencies (higher usage frequency corresponds to higher cleavage efficiency). Note that the Nextera XT dataset had higher total coverage than the Nextera Flex/DNA Prep and therefore, the bins are set higher for that dataset. “LC regions” correspond to the tagmentation sites found in regions yielding low coverage when sequenced using Nextera XT, but the same regions were also analyzed in the Nextera Flex/DNA prep dataset.

Coverage is an indirect measure of the tagmentation bias. To obtain a more direct measure, we analyzed the Nextera XT coverage bias problem on the level of transposase cleavage events, defined by the sequence read starts. To improve resolution, data from laboratories using the same type of Nextera library preparation kit were merged. By analyzing the sequences surrounding the read start position in the reference genome, the average base compositions for the transposase binding sites could be determined for both Nextera Flex/DNA Prep and Nextera XT. The Nextera tagmentation site was highly symmetrical with a central AT-rich region and the average base composition pattern of Nextera XT tagmentation sites looked very similar to that of Nextera Flex/DNA Prep (Figure 5A). The right-hand part of this consensus is seen in base composition plots in quality control programs such as FastQC (Trivedi et al., 2014), and fastp (Chen et al., 2018). The base composition of the tagmentation sites was also analyzed using sequence logos (Figure 5B). The results indicated that the position of four nucleotides upstream and downstream of the center of the motif (positions 1 and 10 relative to the read start site) is most important for the tagmentation motif. The most frequent bases in these positions were Gs and Cs, respectively. This suggests a mechanism where Gs and Cs in these positions are important for tagmentation efficiency and become limiting in AT-rich regions, thus creating a GC-content-dependent bias.

To investigate this further, we divided the tagmentation sites into groups depending on how efficient they were (i.e., the frequency of reads generated from that site when ultra-deep coverage was used). The GC-content of the core cleavage motif (central 20 bp of the motif) was also calculated. When plotting the average GC-content of the motifs in the different efficiency groups, it became clear that the GC-content of the motif was increasing as the efficiency of the sites increased (Figure 5C). The mean of each frequency bin was significantly larger than the mean in the preceding frequency bin and the mean of the LC-regions was significantly lower than the smallest frequency bin (*t-*tests, *p* value < 0.0001 for all tests). The same pattern was seen for both Nextera XT and Nextera Flex/DNA Prep. We also extracted all tagmentation sites present in the previously defined “low coverage regions” (defined based on Nextera XT coverage data). We did this for both Nextera XT and Nextera Flex/DNA Prep. The number of tagmentation events was strongly reduced in Nextera XT data, but the ultra-high coverage made it possible to extract sufficient reads starting in these regions to perform the analysis for Nextera XT as well. For both Nextera XT and Nextera Flex/DNA Prep, the low coverage regions contained tagmentation sites with very low average GC-content. This further supports a mechanistic model where low coverage is caused by the lack of efficient tagmentation sites, which, in turn, is a consequence of low amounts of the Gs and Cs that are crucial for cleavage efficiency. Notably, Nextera Flex/DNA Prep also depends on Gs and Cs for cleavage efficiency but is still able to cleave these sites at a sufficient rate to avoid coverage bias problems. Thus, there seems to be a property of the Nextera Flex/DNA Prep that facilitates cleavage of these low-efficiency sites, which is lacking in Nextera XT.

### Extrapolation to other bacterial species

Finally, we explored additional datasets from other bacterial species to get a broader perspective of the conclusions. To illustrate the magnitude of the bias problem in those datasets, three types of graphs previously used were selected. These were (i) the histogram of the occurrence of the reference genome *k*-mers in the raw data (same as Figure 3C upper part), (ii) the corresponding GC-content plot of the *k*-mer bins (same as Figure 3C lower part), and (iii) the number of contigs generated at different coverages (same as Figure 1A).

**Figure 6:**
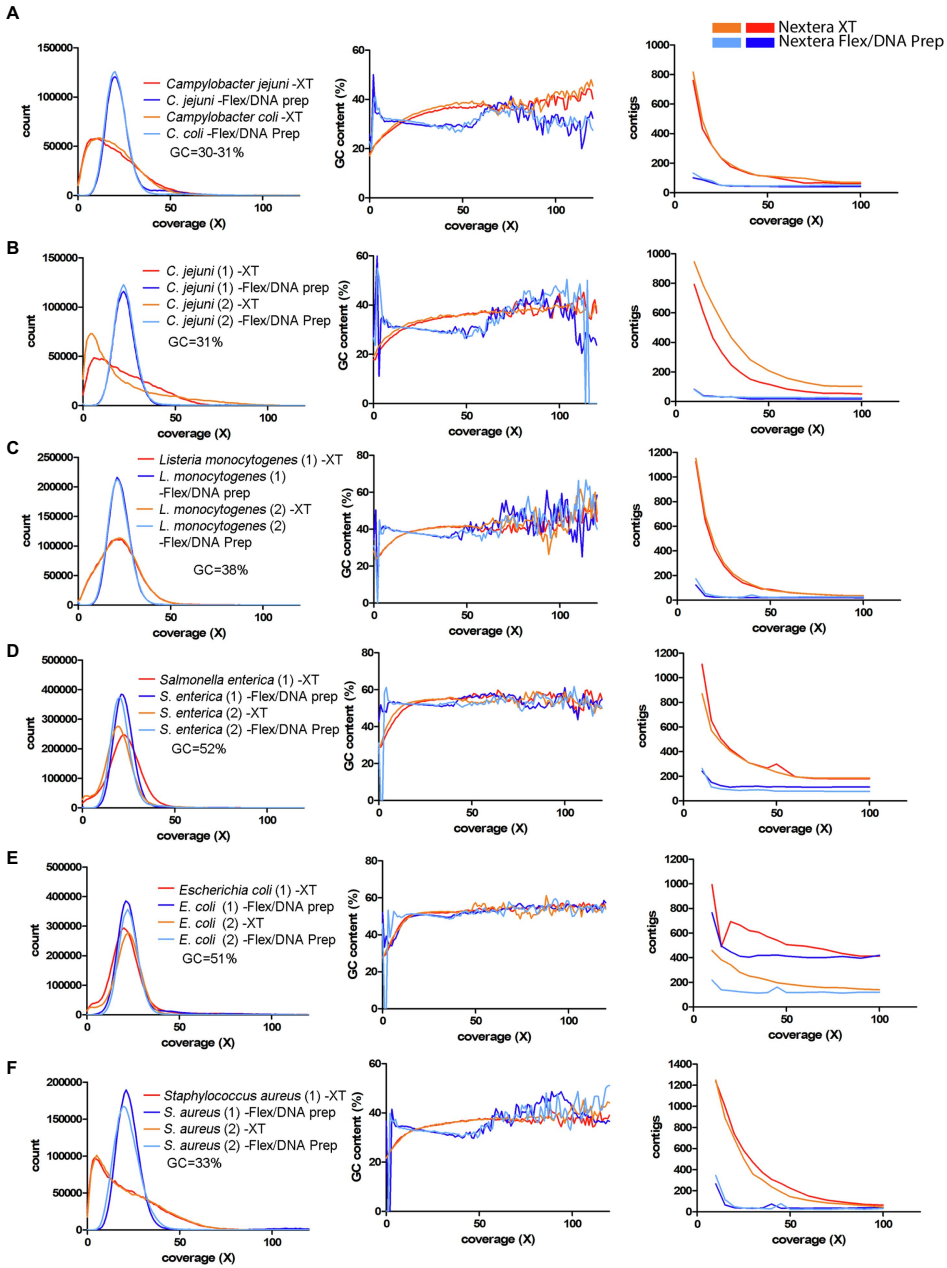
Coverage bias, connection to GC-content, and consequences on assembly efficiency for data produced with Nextera XT and Nextera Flex/DNA Prep in external datasets. The data have been colored to indicate the type of library prep kit used (Nextera XT orange/red, Nextera Flex/DNA Prep light blue/dark blue). Left column represents histograms over the depth in the raw data (downsampled to 50x) of the *k*-mers present in the reference sequence. Middle column represents the corresponding average GC-content of the *k*-mers in each histogram bin. The right column represents the number of contigs generated by SPAdes assembler after filtering short and low coverage contigs. **(A)** EURL-*Campylobacter* PT 28 data including *Campylobacter jejuni* and *C. coli*. **(B–D)** Data from an interlaboratory comparison made by BfR, Germany (Uelze et al., 2020a). Two strains each, of *C. jejuni*, *L. monocytogenes,* and *Salmonella enterica*. **(E,F)** Data from a study by Sato et al. (2019). Two strains each, of *Escherichia coli* and *Staphylococcus aureus*.

First, the *C. coli* strain from the EURL-*Campylobacter* PT 28 (Eurl-Campylobacter, 2020) was analyzed and plotted together with the *C. jejuni* strain from the same test. The data showed that the *C. coli* strain behaved very similarly to the *C. jejuni* strain (Figure 6A). Next, the data from the interlaboratory WGS comparison, performed in 2019 by the German federal institute for risk assessment (BfR; Uelze et al., 2020a), were analyzed. The strains used in this study were two *C. jejuni* strains, two *Listeria monocytogenes* strains, and two *Salmonella enterica* strains. For simplicity, one representative laboratory for Nextera XT and one for Nextera Flex/DNA Prep were chosen from this dataset for the analysis. The data from the *C. jejuni* strains displayed a strong GC-dependent coverage bias, similar to the *Campylobacter* data of this study (Figure 6B). Both for *L. monocytogenes* and *S. enterica*, the *k*-mer coverage distributions were closer to normal distribution compared to *Campylobacter*, but there was a fraction of the genome that generated low coverage, and this was connected to low GC-content (Figures 6C,D). The effect was stronger for *Listeria*, which has an average GC-content that lies in-between *Campylobacter* and *Salmonella* (Figure 6C). The genome assembly process was strongly affected for all three species in this dataset (Figures 6B–D).

In the dataset from Sato et al. (2019), which included *E. coli* and *S. aureus*, the *E. coli* data showed a comparatively low level of coverage bias-related problem (Figure 6E). As for *Salmonella*, the regions that were affected had lower GC-content. The assembly efficiency was also affected but less than the previously analyzed species. For *S. aureus*, the effect of coverage bias was strong, similar to that of *Campylobacter* (Figure 6F).

**Figure 7:**
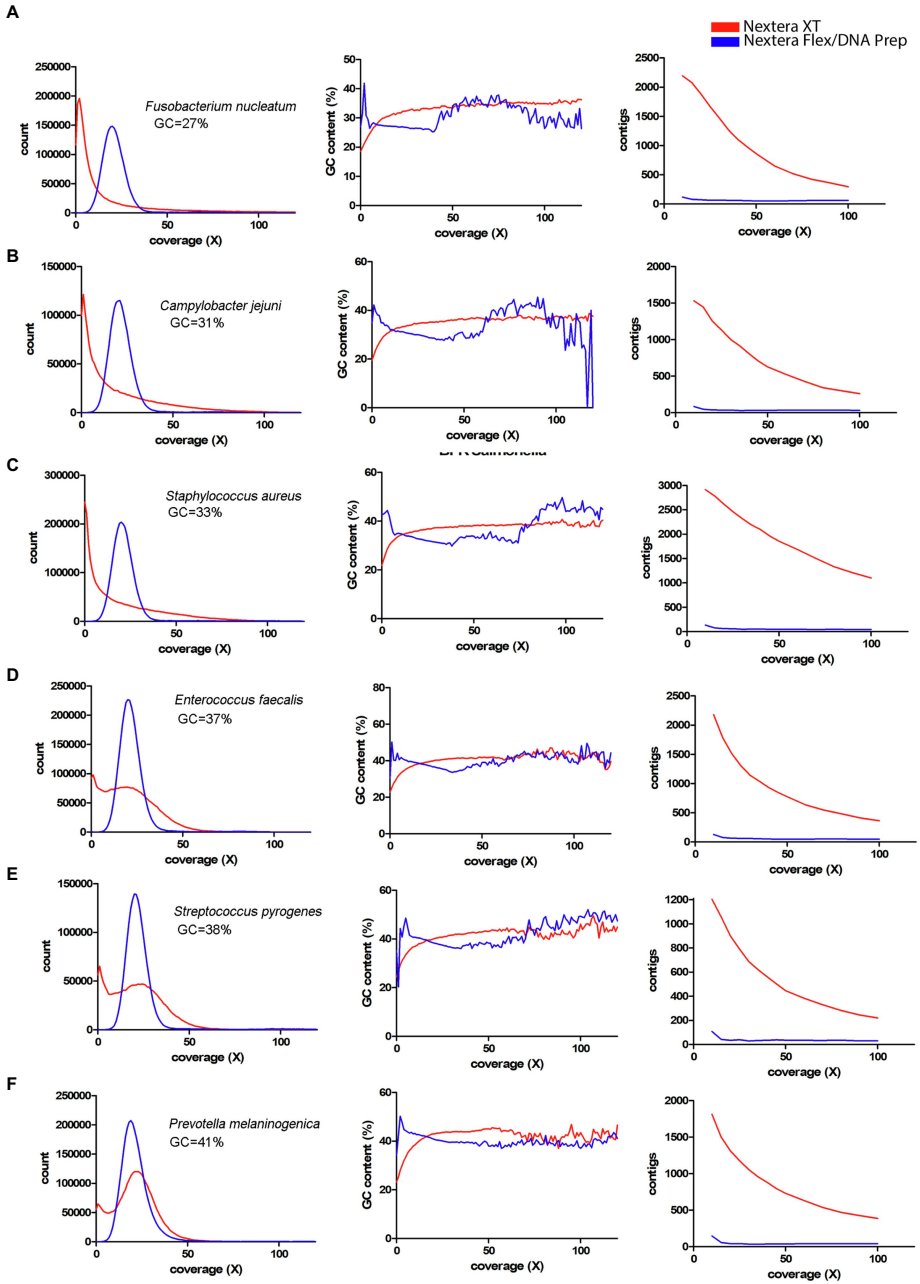
Coverage bias, connection to GC-content, and consequences on assembly efficiency for data produced with Nextera XT and Nextera Flex/DNA Prep in external datasets. The data have been colored to indicate the type of library prep kit used (Nextera XT red, Nextera Flex/DNA Prep blue). Left column represents histograms over the depth in the raw data (downsampled to 50x) of the *k*-mers present in the reference sequence. Middle column represents the corresponding average GC-content of the *k*-mers in each histogram bin. The right column represents the number of contigs generated by SPAdes assembler after filtering short and low coverage contigs. **(A–F)** Data from Seth-Smith et al. (2019) representing bacterial species with low GC-content.

**Figure 8:**
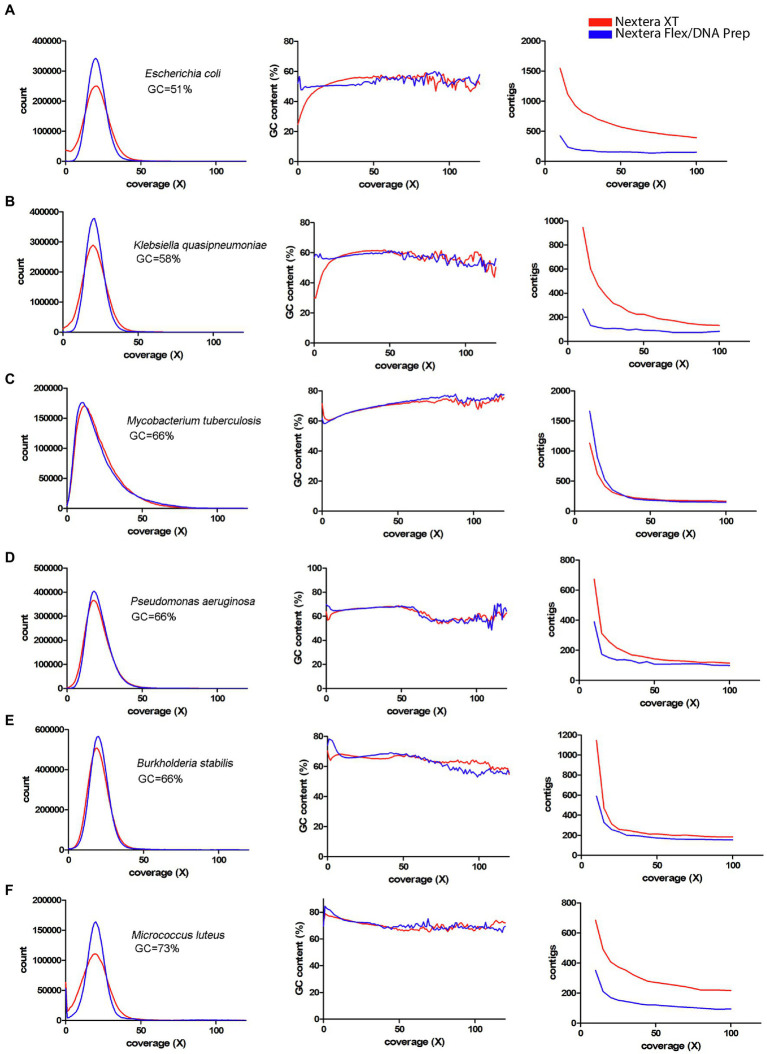
Coverage bias, connection to GC-content, and consequences on assembly efficiency for data produced with Nextera XT and Nextera Flex/DNA Prep in external datasets. The data have been colored to indicate the type of library prep kit used (Nextera XT red, Nextera Flex/DNA Prep blue). Left column represents histograms over the depth in the raw data (downsampled to 50x) of the *k*-mers present in the reference sequence. Middle column represents the corresponding average GC-content of the *k*-mers in each histogram bin. The right column represents the number of contigs generated by SPAdes assembler after filtering short and low coverage contigs. **(A–F)** Data from Seth-Smith et al. (2019) representing bacterial species with medium or high GC-content.

Since a major factor affecting the impact of the coverage bias is the average GC-content, a panel of species representing different GC-contents sequenced by Seth-Smith et al. (2019) was used (Figures 7, 8). From these figures, it is obvious that species with low GC-content are affected globally over the whole genome, whereas species with more neutral GC-content are affected only in certain regions with low GC-content. The bacteria with high GC-content displayed no or very little difference between Nextera XT and Nextera Flex/DNA Prep. Notably, extremely high GC-content once again affected the sequence quality from Nextera XT, but not specifically in AT-rich regions, rather in GC-rich regions.

## Discussion

As WGS is becoming the new standard for bacterial high-resolution typing, it is under the scope of accreditation in many laboratories. Participation in PTs and other interlaboratory comparisons is useful for the implementation of a method and is requested by accreditation bodies. Reports from such tests tend to show that laboratories using Nextera XT for library preparation perform less well compared to other laboratories (Uelze et al., 2020a). This was also evident in PT 28 organized by the EURL-*Campylobacter* in 2020 for sequencing of *Campylobacter* (Eurl-Campylobacter, 2020). In the process of compiling the report for this PT and providing feedback to individual participants, we realized that there was a need to clarify the consequences of using Nextera XT and to get a better understanding of the underlying mechanism of the coverage bias problem. It also seemed valuable to improve the cross-species comparisons to aid laboratories to judge if the Nextera XT problems are of their concern. Thus, the aim of this study was to explore and illustrate this issue to laboratories performing bacterial WGS, to ultimately increase the quality of sequence output and downstream applications such as high-resolution typing methods used in surveillance and outbreak investigations.

Coverage bias problems coupled to Nextera XT have been reported in previous studies. The earliest reports linked high GC-content regions to lower coverage. These studies were made on HLA genes (Lan et al., 2015) and *M. tuberculosis* (Tyler et al., 2016) and did not observe any problems with regions having low GC-content. However, this can be explained by the fact that they were performed on target sequences with high GC-content (60–66% GC). The major GC-bias effects of Nextera XT become visible when sequences with low GC-content are analyzed. This was revealed in a series of reports in 2019 where Seth-Smith et al. compared Nextera XT with other library preparation kits using several different bacterial species. There, it was reported that libraries prepared with Nextera XT showed GC-dependent coverage bias and must be sequenced at a much higher depth to cover the whole genome (Seth-Smith et al., 2019). In a metagenomic study by Grützke et al., Nextera XT was shown to distort the mock community in a GC-content-dependent manner (Grutzke et al., 2019). Likewise, Sato et al. concluded that Nextera XT had problems with low GC-content genomes, both in WGS applications and in the sequencing of metagenomic mock communities (Sato et al., 2019). Another report showed that Nextera XT had problems in detecting regions of the O-antigen in *Salmonella* spp. characterized by low GC-content (Uelze et al., 2020b). Furthermore, in a recent study by Gunasekera et al., Nextera XT coverage bias was found in *E. coli* (Gunasekera et al., 2021), although the authors argued that the benefit of using Nextera Flex/DNA Prep instead of Nextera XT was modest when sequencing the GC-neutral species *E. coli*.

Here, we replicate the main findings of these reports using the *Campylobacter* PT 28 data and make an in-depth analysis of the underlying mechanism for the coverage bias. By examining the bias using frequencies of tagmentation sites instead of read coverage, we could link the efficiency of tagmentation to G and C residues in the tagmentation motif. This provides a more direct explanation of the GC-dependent coverage bias of Nextera XT. Interestingly, a similar link between G and C residues and tagmentation site efficiency was seen in Nextera Flex/DNA Prep, which do not suffer from extensive coverage bias. That is probably due to the fact that the bead-linked transposases of Nextera Flex/DNA Prep first bind the DNA at high efficiency tagmentation sites, and then retain the DNA molecule and present the neighboring regions, including low-efficiency tagmentation sites, to nearby transposases. Likely, this will effectively reduce the impact of dissimilar efficiencies at different tagmentation sites.

We also wanted to clarify the consequences of the coverage bias when sequencing different species. Our compilation clearly shows that the Nextera XT coverage bias problem has a huge effect across the entire genome for low GC-content bacteria such as *Campylobacter*, *Staphylococcus,* and *Listeria*. Even though the usage of very high coverage can overcome many of the problems associated with this, the quality will remain lower than if using Nextera Flex/DNA Prep or another library prep without coverage bias. The fluctuating coverage might also compromise analysis and filtration steps that are based on coverage data. This includes using low coverage as an indicator to filter out contaminations from other species and carry over sequences between sequencing runs, filter out low-quality SNPs, and estimate the copy number of genetic amplifications and plasmids. There is also a clear risk that regions of particular interest will be lost in the WGS analysis if they contain zones with low GC-content.

Bacteria with neutral GC-content, such as *Salmonella*, *Escherichia,* and *Klebsiella*, are still affected by the coverage bias, but the problem is associated with a smaller fraction of the genome with a low GC-content instead of a genome-wide effect. This gives more fragmented genome assemblies and loss of specific genes and regions with low GC-content. For high GC-content organisms such as *Mycobacterium* and *Pseudomonas*, the difference between Nextera XT and Nextera Flex/DNA Prep is hardly visible. Low coverage regions in these species are fewer and are rather associated with a very high GC-content.

In the end, we recommend laboratories performing bacterial WGS typing activities to carefully weigh the advantages and disadvantages if they decide to use the Nextera XT library preparation kit since it will demand higher sequencing depth and still negatively affect downstream analysis when sequencing bacteria with low or neutral GC-content.

## Data availability statement

The datasets presented in this study can be found in online repositories. The names of the repository/repositories and accession number(s) can be found at: https://www.ebi.ac.uk/ena, PRJEB45600.

## Author contributions

BS conceived and performed most of the bioinformatics in the study and drafted the manuscript and the figures. ÁÁ performed cgMLST analysis. LM contributed to the analysis of the BfR dataset. ÁÁ, JS, BS, and HS organized and analyzed PT 28. All authors contributed to the article and approved the submitted version.

## Funding

This work was financially supported by the Swedish Foundation for Strategic Research and the European Union.

## Conflict of interest

The authors declare that the research was conducted in the absence of any commercial or financial relationships that could be construed as a potential conflict of interest.

## Publisher’s note

All claims expressed in this article are solely those of the authors and do not necessarily represent those of their affiliated organizations, or those of the publisher, the editors and the reviewers. Any product that may be evaluated in this article, or claim that may be made by its manufacturer, is not guaranteed or endorsed by the publisher.

## Footnotes

^1^ https://github.com/BoSegerman/tngs

^2^ https://github.com/tseemann/snippy

^3^ http://weblogo.threeplusone.com/create.cgi
